# Synthesis and AChE Inhibitory Activity of Novel Thiazolylhydrazone Derivatives

**DOI:** 10.3390/molecules24132392

**Published:** 2019-06-28

**Authors:** Derya Osmaniye, Begüm Nurpelin Sağlık, Ulviye Acar Çevik, Serkan Levent, Betül Kaya Çavuşoğlu, Yusuf Özkay, Zafer Asım Kaplancıklı, Gülhan Turan

**Affiliations:** 1 Department of Pharmaceutical Chemistry, Faculty of Pharmacy, Anadolu Universty, 26470 Eskişehir, Turkey; 2 Doping and Narcotic Compounds Analysis Laboratory, Faculty of Pharmacy, Anadolu Universty, 26470 Eskişehir, Turkey

**Keywords:** thiazolylhydrazone, acetylcholinesterase, butyrylcholinesterase, enzyme inhibition, docking

## Abstract

Alzheimer’s disease (AD) is the most common of the degenerative brain diseases and is described together with the impairment of cognitive function. Patients with AD lose the capability to code new memories, and life conditions are extremely difficult. The development of new drugs in this area continues at a great pace. A novel series of thiazole-piperazine hybrids, aimed against Alzheimer’s disease (AD), have been synthesized. The structure identification of synthesized compounds was elucidated by ^1^HNMR, ^13^C-NMR, and LCMSMS spectroscopic methods. The inhibitory potential of the synthesized compounds on cholinesterase enzymes was investigated. The compounds **3a**, **3c** and **3i** showed significant inhibitory activity on the acetylcholinesterase (AChE) enzyme. On the other hand, none of the compounds showed significant inhibitory activity on the butyrylcholinesterase (BChE) enzyme. In addition to enzyme inhibition studies, enzyme kinetic studies were performed to observe the effects of the most active inhibitor compounds on the substrate–enzyme relationship. In addition to in vitro tests, docking studies also indicated that compound **3c** potentially acts as a dual binding site AChE inhibitor.

## 1. Introduction

Alzheimer’s disease (AD) is a condition that rapidly threatens public health, with an increasing number of affected individuals as the world population increases. Currently, there are 35 million people with AD dementia in the world, and this number will increase to approximately 100 million globally by 2050 if treatments are not found [1]. There are two main hypotheses about the main factors and causes contributing to AD: the cholinergic and β-amyloid hypotheses. For each hypothesis, the key to slowing the progression of the disease is either to reduce cholinergic activity or to reduce the accumulation of β-amyloid plaques as a major pathological sign, respectively [2,3,4,5]. There are also other major factors of AD that must be considered. One of them is the hyperphosphorylation of tau protein, which results in insoluble tau deposits that affect microtubule stability and possibly increase the toxicity of β-amyloid. There is also concern over reactive oxygen species (ROS) and their promotion of the disease pathology by affecting oxidative stress, which has been found to thoroughly relate to amyloid plaques [6].

Interestingly, the new data suggest that the decrease in cholinergic activity and the accumulation of β-amyloid plaques are related. One relationship presented is the evidence that acetylcholinesterase (AChE) increases the deposition of β- amyloid plaques, which in turn increases the expression of AChE. Significantly, brain levels of another enzyme that hydrolyses ACh, butyrylcholinesterase (BuChE), show progressive and significant increases in AD [7,8].

The pharmacological effects of AChE inhibitors are the inactivation of enzyme activity, resulting in the increased stimulation of postsynaptic cholinergic receptors in peripheral and/or central nervous systems and the depletion of synaptic acetylcholine (ACh). AChE inhibitors can be divided into two groups according to their mode of action: irreversible and reversible. Reversible, noncompetitive or competitive, inhibitors play a leading role in the pharmacotherapy of AD symptoms. They show their therapeutic effects via maintaining the ACh level through retarding the rate of its hydrolysis. Consequently, commonly well-tolerated drugs compensate for the loss of working brain cells and improve cholinergic neurotransmission in the anterior brain regions. Above and over AD, reversible AChE inhibitors of different chemical structures have significant pharmacological application in the treatment of neurological disorders appearances such as Parkinson’s disease dementia, Lewy bodies, and myasthenia gravis, and also as prophylactics against nerve agent intoxication [9].

Among all well-known AChE inhibitors used medically, four different sub-classes can be used to simplify comparison relative to their structural origins and functional groups.

Donepezil and Tacrine best signify examples of synthetic AChE inhibitors with unique structural nuclei, such as *N*-benzyl piperidine and acridine, respectively. However, Tacrine (1,2,3,4-tetrahydroacridin-9-amine) has since been removed from the market due to adverse effects, including hepatotoxicity in a significant percentage of patients. On the other hand, Huperzin A and Galantamine are examples of natural products that display strong, selective and reversible inhibitory activity against AChE. Physostigmine and its derivative Rivastigmin are examples of carbamate-type AChE inhibitors [8,10].

A thiazole derivative, Acotiamide, has been reported to be a new selective AChE inhibitor for the treatment of functional dyspepsia [11]. This development caused the enhancement of studies on the structure of thiazole as an AChE inhibitor [12,13,14,15,16]. Some 2-Aminothiazole derivatives were also patented in 2014 and 2015 that showed inhibitory activity against AChE [17,18].

**Figure 1:**
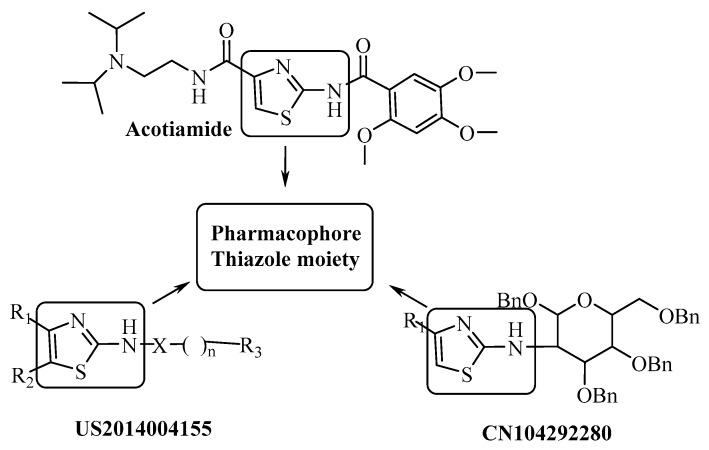
2-amino-4-substituted thiazole as a pharmacophore for acetylcholinesterase (AChE).

Although the new thiazole derivatives have different substituents, the 2-amino-4-substituted thiazole as a common structure has supported us in this study (Figure 1).

**Figure 2:**
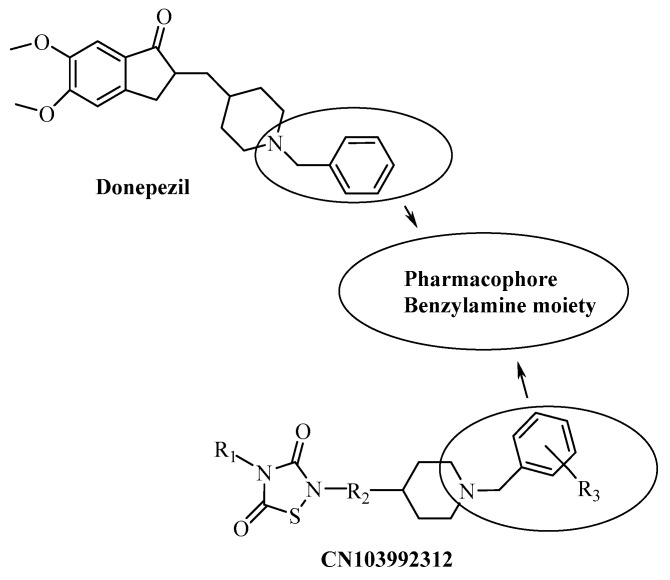
Some AchE inhibitors, including a benzylamine moiety.

On the other hand, there are many studies on novel AChE inhibitor compounds for use as donepezil analogues, having a pharmacophore benzylamine moiety as a basic center [19,20,21,22,23,24] (Figure 2). The compounds we synthesized in this study included a benzylamine moiety and thiazole bridges with aromatic groups at both ends [7,25,26].

Monoamine oxidase (MAO) enzymes play an important role in the regulation of different biological processes, and thus they are a significant target in drug design for the treatment of psychiatric and neurological diseases. Especially for symptoms of Parkinson’s and Alzheimer’s diseases, MAO-B (Monoamine oxidase B) inhibitors are generally used. On the other hand, MAO-A (Monoamine oxidase A) inhibitors are clinically used as antidepressants and anxiolytics. Therefore, in the treatment of Alzheimer’s disease, the evaluation of monoamine oxidase enzyme inhibitory activity is generally preferred along with cholinesterase inhibition assays [27,28,29]. 

**Figure 3:**
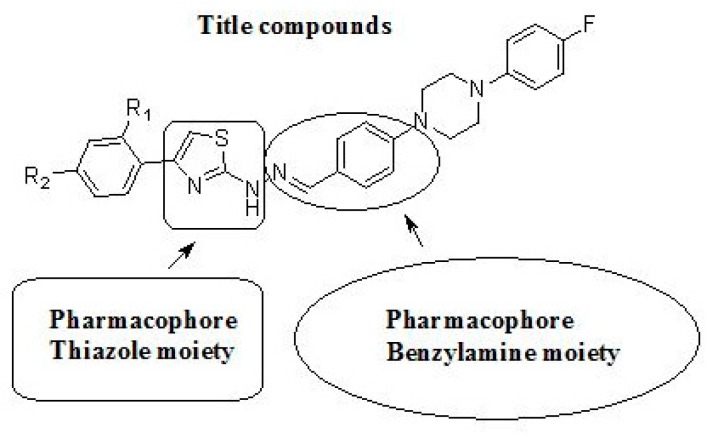
General structure of target compounds.

Based on these findings, we became interested in the biological evaluation of 2-aminothiazoles as anticholinesterase agents. Herein, we describe the synthesis of novel thiazole derivatives bearing a benzylamine moiety and focus on their anticholinesterase effects on AChE (Figure 3).

## 2. Results and Discussion

### 2.1. Chemistry

**Scheme 1:**
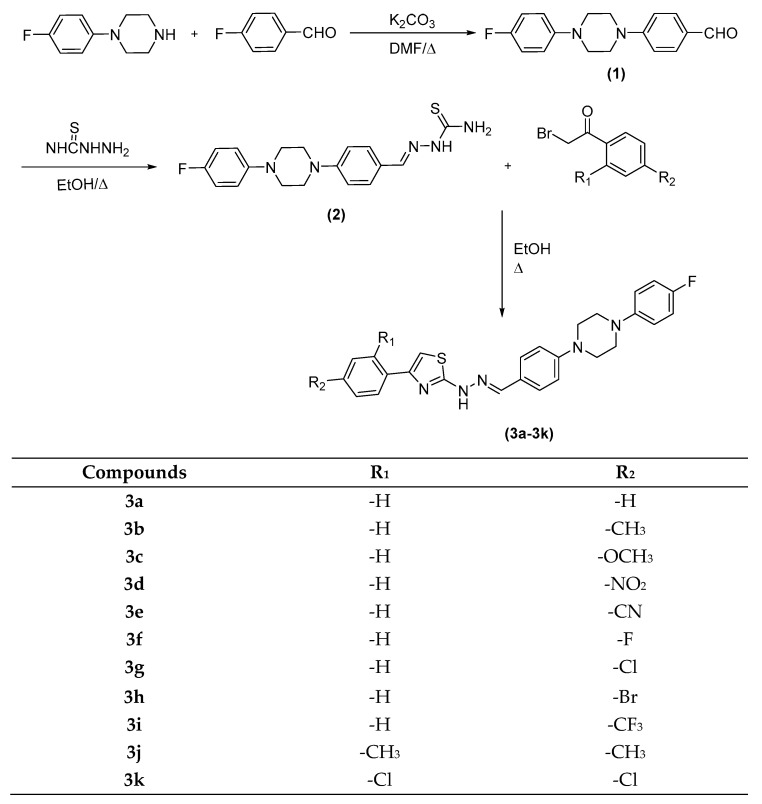
Synthesis of the target compounds **(3a–k)**.

The compounds **3a**–**k** were synthesized as outlined in Scheme 1. Firstly, 4-(4-(4-fluorophenyl)piperazin-1-yl)benzaldehyde was synthesized by a reaction of 1-(4-fluorophenyl)piperazine and 4-Fluorobenzaldehyde. Then, aldehyde (**1**) reacted with thiosemicarbazide, which was performed in ethanol with a catalytic amount of CH_3_COOH to provide 2-(4-(4-(4-fluorophenyl)piperazin-1-yl)benzylidene)hydrazine-1-carbothioamide (2) in good yields. Finally, target compounds (**3a**–**l**) were prepared through ring closure reaction using obtained thiosemicarbazide (**2**) and an appropriate 2-bromoacetophenone. The final compounds were purified, and their structures were characterized by spectroscopic methods (^1^H-NMR, ^13^C-NMR, and LCMSMS). In the ^1^H NMR spectrum, 1,4-disubstituted benzene protons had doublet peaks between 7.52 ppm and 7.55 ppm. Fluorophenyl hydrogens and disubstitue phenyl hydrogens gave multiplet peaks between 6.97 ppm and 7.12 ppm. Thiazole hydrogens had singlet peaks between 7.09 ppm and 7.68 ppm. Hydrazone protons gave singlet peaks between 7.94 ppm and 7.97 ppm. In the ^13^C NMR spectrum, aliphatic peaks belonging to substituents were observed between 21 ppm and 55 ppm. Aromatic carbons were gained between 102 ppm and 169 ppm. Carbonyl carbon gave a peak over 168 ppm. All masses were in accordance with the estimated M+H values. Structure elucidations of the final compounds were performed by IR, ^1^H-NMR, ^13^C-NMR, and MS spectroscopic methods (see Supplementary Materials)

### 2.2. Anticholinesterase Activity Assay

To determine the potential interest of the thiazole-piperazine synthesized, AChE and butyrylcholinesterase (BChE) inhibitory potency was evaluated according to a modified Ellman method with commercially obtainable donepezil as the reference standard. Initially, all the obtained compounds were tested at 10^−3^ M and 10^−4^ M concentrations and the ChE inhibitory results are outlined in Table 1. With respect to the activity results, it was determined that most of the tested compounds showed better inhibitory activity against AChE than BChE activity. Compounds **3a**, **3c** and **3i** indicated more than 50% activity at the 10^−4^ M concentration. Then, the IC_50_ values of the active compounds were determined using 10^−3^–10^−9^ M concentrations against AChE, along with the reference drug donepezil (Table 1). The IC_50_ values of the compounds **3a**, **3c** and **3i** were calculated as 0.0496 ± 0.002 µM, 0.0317 ± 0.001 µM and 0.2158 ± 0.010 µM, respectively, for AChE. According to the results of activity tests, some general results can be listed as follows. The compounds **3a**, **3c** and **3i** contain the non-substituted, methoxy and trifluoromethyl substituents on the phenyl ring, respectively. The most potent AchE inhibitory activity was observed for the compounds with methoxy at the para position of the benzene ring (**3c**), with IC_50_ values of 0.0317 µM.

### 2.3. Monoamine Oxidase Activity Assay

In order to investigate the monoamine oxidase enzyme inhibitory effects of synthesized compounds, MAO-A and MAO-B enzyme activities were performed by using a fluorometric inhibition method. Moclobemide and selegiline were used as standard drugs for MAO-A and MAO-B enzymes, respectively. Test compounds and standard drugs were used in the same concentrations as in AChE and BChE enzyme activity assays. In Table 2, MAO enzyme inhibitory results are given. According to these findings, none of the tested compounds displayed remarkable inhibition potency against the MAO-A enzyme. It was seen that synthesized compounds showed better inhibition profile against MAO-B that MAO-A. The obtained compounds showed generally moderate activity against the MAO-B enzyme. Compounds **3a**, **3c** and **3d** showed inhibition profiles with 2.107 ± 0.086 µM, 1.015 ± 0.042 µM and 5.204 ± 0.153 µM IC_50_ values, respectively.

### 2.4. Enzyme Kinetic Studies

**Figure 4:**
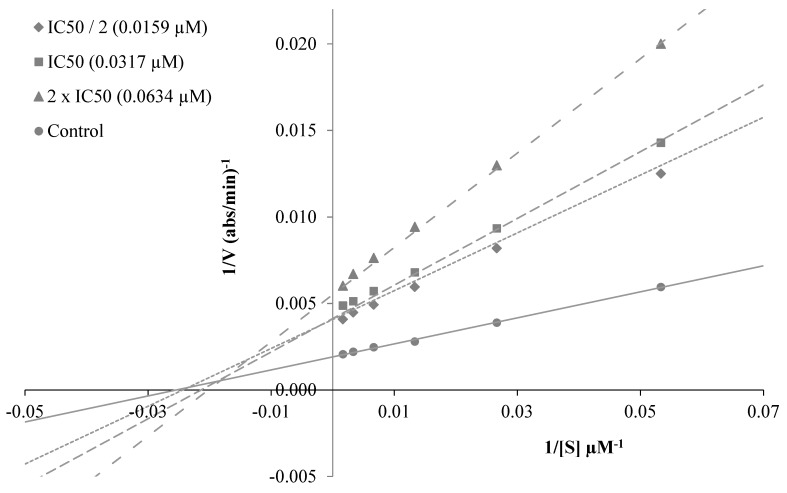

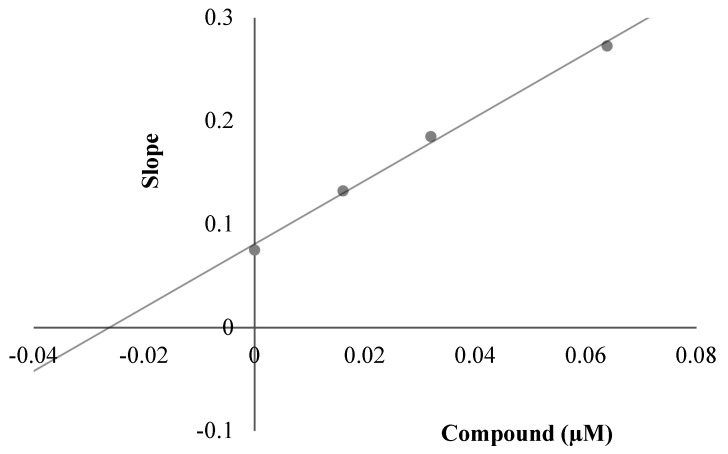
(**A**) Lineweaver–Burk plots for the inhibition of AChE by compound **3c**. [S], substrate concentration (μM); V, reaction velocity (abs/min)^−1^. (**B**) Secondary plot for the calculation of steady-state inhibition constant (K_i_) of compound **3c**. Ki was calculated as 0.0197 μM.

To decide the mechanism of enzyme inhibition, the kinetic study was performed with the most active compounds, **3a**, **3c** and **3i**. The type of inhibition was estimated by means of linear Lineweaver-Burk lands, and the substrate speed curves in the presence and absence of **3a**, **3c** and **3i** of the strongest compounds were verified in the enzyme kinetics analysis. Compounds used for kinetic study were ready at concentrations of 2 × IC_50_, IC_50_ and IC_50_/2. In the initial velocity measurements, different substrate concentrations, ranging from 600 µM to 18.75 µM, were used for the AChE enzyme. The secondary plots of the Km/Vmax (slope) versus changing concentrations were used to determine Ki (intercept on the x-axis) values of compounds [30]. The graphical analyses of steady-state inhibition data for representative compound **3c** are exhibited in Figure 4. Reversible inhibition comprises of the ensuing subtypes: competitive, uncompetitive, noncompetitive or mixed-type. Such inhibitions can be determined using Lineweaver–Burk plots. According to the Lineweaver–Burk graphics, mixed type inhibition can be allowed if the lines do not cross the x or y axis at the same point. Based on the gained plot, a mixed type of inhibition by compound **3c** was established. That is, the compound can bind to the enzyme–substrate complex or to the free enzyme. It also means that the compound is a reversible inhibitor; reversible compounds can be bound to the enzyme by non-covalent interactions such as ionic bonds, hydrophobic interactions and hydrogen bonds deprived of any chemical bonding or reaction with the enzyme. These interactions take place quickly and can be easily reversed. For that reason, the enzyme–inhibitor complex is rapidly degraded immediately before irreversible inhibition. With this feature, these reversible inhibitors exhibit a lower risk of side effects than irreversible inhibitors. As a result, enzyme kinetics studies have shown the biological importance of compound **3c** owing to its mixed type forces, as opposed to reversible enzyme inhibitors.

### 2.5. Molecular Docking Studies

Docking studies were performed in order to gain more insight into the binding mode of compound **3c** to the AChE enzyme. Studies were carried out by using the X-ray crystal structure of Homo sapiens AChE (hAChE PDB ID:4EY7) [31], obtained from the Protein Data Bank server (www.pdb.org).

**Figure 5:**
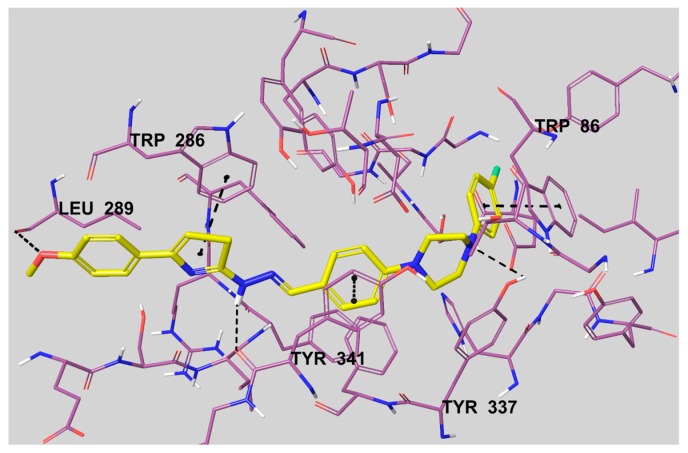
The interacting mode of compound **3c** in the active region of AChE. The inhibitor, colored with yellow, and the important residues, colored with purple, in the active site of the enzyme are presented by a tube model.

The 5,6-dimethoxy indanone moiety binds to the peripheral anionic site (PAS) of the enzyme active region by interacting with Trp286 and Phe295 amino acid residues. The rest of the structure, a benzylpiperidine group, binds to the catalytic anionic site (CAS) by interacting with Trp86 [31]. The docking pose of compound **3c** is presented in Figure 5. It can be seen from this figure that compound **3c** settles in the CAS and PAS regions of the enzyme active site, in a similar position to donepezil. This compound could bind to both PAS and CAS by interacting with Trp286 and Trp86 amino acids, respectively.

According to Figure 5, the thiazole ring creates a π-π interaction with the indole of Trp286. The other π-π interaction is seen between the 4-fluorophenyl ring and the indole of Trp86. The last similar interaction is observed between the phenyl ring, near to the piperidine ring, and the phenyl of Tyr341. There are three hydrogen bonds besides these mentioned interactions. The first of the hydrogen bonds is seen between the oxygen atom of the 4-methoxyphenyl moiety and the carbonyl of Leu289. Also, it is understood that the nitrogen atoms of the imine group and the piperidine ring form a basic center. The amino of the imine group establishes a hydrogen bond with the carbonyl of Tyr341. The last hydrogen bond is observed between the nitrogen atom of the piperidine ring and the hydroxyl of Tyr337.

### 2.6. Prediction of Blood–Brain Barrier (BBB) Permeability

It is known that drugs that specifically target the central nervous system (CNS) have to pass the blood–brain barrier (BBB). Thus, penetration to the BBB is very important for CNS drug candidates, and should be clarified as early as possible in the drug discovery studies [32]. Therefore, the BBB permeability of the synthesized compounds (**3a**–**k**) was calculated by a CBLigand-BBB prediction server [33]. This online predictor practices two different algorithms, AdaBoost and Support Vector Machine (SVM), combining with four different fingerprints, employed to predict if a compound can pass (+) or cannot pass (−) the BBB. Predictor scores are higher than 0 if a compound can pass the BBB. Calculations for all compounds resulted in BBB (+), which is essential for AchE inhibitors to display their biological activity.

## 3. Materials and Methods

### 3.1. Chemistry

All chemicals used in the syntheses were purchased either from Merck Chemicals (Merck KGaA, Darmstadt, Germany) or Sigma-Aldrich Chemicals (Sigma-Aldrich Corp., St. Louis, MO, USA). The reactions and the purities of the compounds were observed by thin layer chromatography (TLC) on silica gel 60 F254aluminum sheets obtained from Merck (Darmstadt, Germany). Melting points of the synthesized compounds were recorded by MP90 digital melting point apparatus (Mettler Toledo, Ohio, USA) and were presented as uncorrected. ^1^H NMR and ^13^C NMR spectra were recorded by a Bruker 300 MHz and 75 MHz digital FT-NMR spectrometer (Bruker Bioscience, Billerica, MA, USA) in DMSO-*d*_6_, respectively. In the NMR spectra, splitting patterns were designated as follows: s: singlet; d: doublet; t: triplet; m: multiplet. Coupling constants (*J*) were reported as Hertz. LC-MS-MS studies were performed on a Schimadzu, 8040 LCMSMS spectrophotometer (Shimadzu, Tokyo, Japan).

#### 3.1.1. Synthesis of 4-(4-(4-fluorophenyl)piperazin-1-yl)benzaldehyde (1)

To a stirred solution of 1-(4-fluorophenyl)piperazine (2.23 g, 0.0124 mol) in DMF (20 mL), 4-fluorobenzaldehyde (1.34 mL, 0.0124 mol) was added, followed by potassium carbonate (1.71 g, 0.0124 mol). The resulting mixture was heated to reflux at 160 °C. After the completion of the reaction, the mixture was poured into iced water after cooling, and the precipitated product was washed with water, dried, and recrystallized from ethanol.

#### 3.1.2. Synthesis of 2-(4-(4-(4-fluorophenyl)piperazin-1-yl)benzylidene)hydrazine-1-carbothioamide (2)

A mixture of 4-(4-(4-fluorophenyl)piperazin-1-yl)benzaldehyde (**2**) (3.18 gr, 0.0112 mol), thiosemicarbazide (1.01 gr, 0.0112 mol), and a catalytic quantity of acetic acide (CH_3_COOH) was refluxed in ethanol (50 mL) for 24 h. After the completion of the reaction, the mixture was cooled, the precipitated product was filtered, washed with cold ethanol, dried, and recrystallized from methanol.

#### 3.1.3. General procedure for the synthesis of target compounds (**3a–j**)

To a stirred solution of 2-(4-(4-(4-fluorophenyl)piperazin-1-yl)benzylidene)hydrazine-1-carbothioamide (**2**) (0.84 mmol) in EtOH (20 mL), appropriate 2-bromo-1-(2,4-substituephenyl)ethan-1-one derivatives (0.84 mmol) were added, and then refluxed for 10 h. The precipitated product was filtered, washed with cold ethanol, dried, and recrystallized from ethanol. 

##### 2-(2-(4-(4-(4-fluorophenyl)piperazin-1-yl)benzylidene)hydrazineyl)-4-phenylthiazole (**3a**)

Yield: 85 %, M.P. = 247.6–249.9 °C, ^1^H-NMR (300 MHz, DMSO-*d_6_*): 3.20–3.22 (m, 4H, piperazine), 3.35–3.38 (m, 4H, piperazine), 6.97–7.09 (m, 6H, fluorophenyl, disubstituephenyl), 7.27 (s, 1H, thiazole), 7.32 (t, 1H, *J* = 5.8 Hz, monosubstituephenyl), 7.41 (t, 2H, *J* = 7.3 Hz, monosubstituephenyl), 7.53 (d, 2H, *J* = 8.8 Hz, disubstituepheyl), 7.86 (d, 2H, *J* = 8.4 Hz, monosubstituephenyl), 7.95 (s, 1H, −N=CH−), 11.93 (s, 1H, −NH). ^13^C-NMR (75 MHz, DMSO-*d_6_*): δ = 48.1 (CH2), 49.5 (CH2), 103.6 (CH), 109.3 (CH), 115.5 (CH), 115.8 (*J_2_* = 21.6 Hz, CH), 118.0 (*J_3_* = 7.4 Hz, CH), 125.3 (C), 126.0 (CH), 128.0 (CH), 129.1 (CH), 135.3 (C), 138.0 (C), 142.2 (CH), 148.2 (C), 151.9 (C), 156.7 (*J_1_*=234.4 Hz, C), 168.9 (C). ESI-MS [M + H]^+^: 458.

##### 2-(2-(4-(4-(4-fluorophenyl)piperazin-1-yl)benzylidene)hydrazineyl)-4-(p-tolyl)thiazole (**3b**)

Yield: 80%, M.P. = 187.5–189.2 °C, ^1^H-NMR (300 MHz, DMSO-*d_6_*): 2.32 (s, 3H, −CH_3_), 3.22–3.23 (m, 4H, piperazine), 3.35–3.37 (m, 4H, piperazine), 7.02–7.07 (m, 6H, fluorophenyl, disubstituephenyl), 7.19–7.22 (m, 3H, thiazole, disubstituephenyl), 7.52 (d, 2H, *J* = 8.8 Hz, disubstituepheyl), 7.74 (d, 2H, *J* = 8.1 Hz, disubstituephenyl), 7.94 (s, 1H, −N=CH−), 11.89 (s, 1H, −NH). ^13^C-NMR (75 MHz, DMSO-*d_6_*): δ = 21.2 (CH_3_), 48.0 (CH_2_), 49.5 (CH_2_), 102.7 (CH), 114.52 (C), 115.5 (CH), 115.8 (*J_2_* = 21.8 Hz, CH), 118.0 (*J_3_* = 7.5 Hz, CH), 125.3 (C), 126.0 (CH), 127.9 (CH), 129.6 (CH), 131.8 (C), 132.6 (C), 137.2 (C), 142.2 (CH), 148.2 (C), 151.9 (C), 157.1 (*J_1_* = 234.7 Hz, C), 168.7 (C). ESI-MS [M + H]^+^: 472.

##### 2-(2-(4-(4-(4-fluorophenyl)piperazin-1-yl)benzylidene)hydrazineyl)-4-(4-methoxyphenyl)thiazole (**3c**)

Yield: 79%, M.P. = 241.7–244.4 °C, ^1^H-NMR (300 MHz, DMSO-*d_6_*): 3.22–3.23 (m, 4H, piperazine), 3.35-3.37 (m, 4H, piperazine), 3.78 (s, 3H, −OCH_3_), 6.96 (d, 2H, *J* = 8.8 Hz disubstituephenyl), 7.02–7.07 (m, 6H, fluorophenyl, disubstituephenyl), 7.09 (s, 1H, thiazole), 7.52 (d, 2H, *J* = 8.7 Hz, disubstituephenyl), 7.55 (d, 2H, *J* = 8.8 Hz, disubstituephenyl), 7.78 (d, 2H, *J* = 8.8 Hz, disubstituephenyl), 7.94 (s, 1H, −N=CH−), 11.88 (s, 1H, −NH). ^13^C-NMR (75 MHz, DMSO-*d_6_*): δ = 48.0 (CH_2_), 49.5 (CH_2_), 55.6 (CH_3_), 101.5 (CH), 114.4 (CH), 115.5 (CH), 115.8 (*J_2_* = 21.8 Hz, CH), 118.0 (*J_3_* = 7.6 Hz, CH), 125.3 (C), 127.3 (CH), 127.9 (CH), 138.9 (C), 142.1 (CH), 148.2 (C), 151.9 (C), 156.8 (*J_1_* = 228.4 Hz, C), 159.2 (C), 168.7 (C). ESI-MS [M + H]^+^: 488.

##### 2-(2-(4-(4-(4-fluorophenyl)piperazin-1-yl)benzylidene)hydrazineyl)-4-(4-nitrophenyl)thiazole (**3d**)

Yield: 84%, M.P. = 236.6–238.9 °C. ^1^H-NMR (300 MHz, DMSO-*d_6_*): 3.28–3.30 (m, 4H, piperazine), 3.40–3.42 (m, 4H, piperazine), 7.05–7.12 (m, 6H, fluorophenyl, disubstituephenyl), 7.55 (d, 2H, *J* = 8.8 Hz, disubstituephenyl), 7.68 (s, 1H, thiazole), 7.97 (s, 1H, −N=CH−), 8.10 (d, 2H, *J* = 9.0 Hz, disubstituepheyl), 8.27 (d, 2H, *J* = 9.0 Hz, disubstituephenyl), 12.06 (s, 1H, −NH). ^13^C-NMR (75 MHz, DMSO-*d_6_*): δ = 47.9 (CH_2_), 49.9 (CH_2_), 108.6 (CH), 115.7 (CH), 116.0 (*J_2_* = 21.9 Hz, CH), 118.6 (*J_3_* = 7.5 Hz, CH), 124.6 (CH), 125.3 (C), 126.8 (CH), 128.0 (CH), 141.2 (C), 142.8 (CH), 146.6 (C), 147.3 (C), 148.9 (C), 151.7 (C), 157.3 (*J_1_* = 235.7 Hz, C), 169.2 (C). ESI-MS [M + H]^+^: 503.

##### 4-(2-(2-(4-(4-(4-fluorophenyl)piperazin-1-yl)benzylidene)hydrazineyl)thiazol-4-yl)benzonitrile (**3e**)

Yield: 77%, M.P. = 251.2–254.8 °C,^1^H-NMR (300 MHz, DMSO-*d_6_*): 3.20–3.22 (m, 4H, piperazine), 3.35-3.36 (m, 4H, piperazine), 7.00–7.06 (m, 6H, fluorophenyl, disubstituephenyl), 7.53 (d, 2H, *J* = 8.8 Hz, disubstituephenyl), 7.58 (s, 1H, thiazole), 7.85 (d, 2H, *J* = 8.5 Hz, disubstituepheyl), 7.97 (s, 1H, −N=CH−), 8.03 (d, 2H, *J* = 8.5 Hz, disubstituephenyl), 12.01 (s, 1H, −NH). ^13^C-NMR (75 MHz, DMSO-*d_6_*): δ = 47.9 (CH_2_), 49.5 (CH_2_), 107.5 (C), 109.9 (CH), 115.5 (CH), 115.8 (*J_2_* = 21.9 Hz, CH), 118.1 (*J_3_* = 7.4 Hz, CH), 119.5 (C), 125.1 (C), 126.6 (CH), 128.0 (CH), 133.1 (CH), 139.3 (C), 142.7 (CH), 148.1 (C), 149.3 (C), 151.9 (C), 156.8 (*J_1_* = 242.1 Hz, C). 169.2 (C). ESI-MS [M + H]^+^: 483.

##### 4-(4-fluorophenyl)-2-(2-(4-(4-(4-fluorophenyl)piperazin-1-yl)benzylidene)hydrazineyl)thiazole (**3f**)

Yield: 75%, M.P. = 250.1–251.9 °C, ^1^H-NMR (300 MHz, DMSO-*d_6_*): δ 3.19-3.22 (m, 4H, piperazine), 3.35–3.38 (m, 4H, piperazine), 7.01–7.06 (m, 6H, fluorophenyl, disubstituephenyl), 7.19–7.26 (m, 3H, thiazole, disubstituephenyl), 7.52 (d, 2H, *J* = 8.8 Hz, disubstituepheyl), 7.90 (dd, 2H, *J_1_* = 5.6 Hz, *J_2_* = 8.1 Hz, disubstituephenyl), 7.95 (s, 1H, −N=CH−), 11.92 (s, 1H, −NH). ^13^C-NMR (75 MHz, DMSO-*d_6_*): δ = 48.0 (CH_2_), 49.5 (CH_2_), 103.4 (CH), 115.5 (CH), 115.8 (*J_2_* = 21.7 Hz, CH), 115.9 (*J_2′_* = 21.3 Hz, CH), 118.0 (*J_3_* = 7.4 Hz, CH), 125.2 (C), 127.9 (CH), 127.9 (*J_3′_* = 7.6 Hz, CH), 131.9 (C), 142.3 (CH), 148.2 (C), 149.9 (C), 151.9 (C), 156.7 (*J_1_* = 233.2 Hz, C), 162.1 (*J_1′_* = 245.1 Hz, C), 168.9 (C). ESI-MS [M + H]^+^: 476.

##### 4-(4-chlorophenyl)-2-(2-(4-(4-(4-fluorophenyl)piperazin-1-yl)benzylidene)hydrazineyl)thiazole (**3g**)

Yield: 73%, M.P. = 236.2–238.8 °C, ^1^H-NMR (300 MHz, DMSO-*d_6_*): 3.20–3.23 (m, 4H, piperazine), 3.35–3.39 (m, 4H, piperazine), 7.02–7.07 (m, 6H, fluorophenyl, disubstituephenyl), 7.34 (s, 1H, thiazole), 7.46 (d, 2H, *J* = 8.6 Hz, disubstituepheyl), 7.53 (d, 2H, *J* = 8.8 Hz, disubstituephenyl), 7.87 (d, 2H, *J* = 8.6 Hz, disubstituephenyl), 7.95 (s, 1H, −N=CH−), 11.94 (s, 1H, −NH). ^13^C-NMR (75 MHz, DMSO-*d_6_*): δ = 47.9 (CH_2_), 49.6 (CH_2_), 104.4 (CH), 115.5 (CH), 115.8 (*J_2_* = 21.9 Hz, CH), 118.1 (*J_3_* = 7.6 Hz, CH), 125.3 (C), 127.7 (CH), 127.9 (CH), 129.1 (CH), 132.3 (C), 134.1 (C), 142.4 (CH), 148.0 (C), 149.1 (C), 151.9 (C), 156.8 (*J_1_* = 234.7 Hz, C), 169.0 (C). ESI-MS [M + H]^+^: 492.

##### 4-(4-bromophenyl)-2-(2-(4-(4-(4-fluorophenyl)piperazin-1-yl)benzylidene)hydrazineyl)thiazole (**3h**)

Yield: 80%, M.P. = 243.2–246.7 °C, ^1^H-NMR (300 MHz, DMSO-*d_6_*): 3.20–3.23 (m, 4H, piperazine), 3.35–3.38 (m, 4H, piperazine), 7.02–7.07 (m, 6H, fluorophenyl, disubstituephenyl), 7.35 (s, 1H, thiazole), 7.53 (d, 2H, *J* = 8.8 Hz, disubstituepheyl), 7.59 (d, 2H, *J* = 8.6 Hz, disubstituephenyl), 7.80 (d, 2H, *J* = 8.6 Hz, disubstituephenyl), 7.95 (s, 1H, −N=CH−), 11.93 (s, 1H, −NH). ^13^C-NMR (75 MHz, DMSO-*d_6_*): δ = 48.0 (CH_2_), 49.5 (CH_2_), 104.5 (CH), 115.5 (CH), 115.8 (*J_2_* = 21.9 Hz, CH), 118.1 (*J_3_* = 7.7 Hz, CH), 120.9 (C), 125.2 (C), 127.9 (CH), 128.0 (CH), 132.0 (CH), 134.5 (C), 142.4 (CH), 148.1 (C), 149.7 (C), 151.9 (C), 156.8 (*J_1_* = 234.4 Hz, C), 169.0 (C). ESI-MS [M + H]^+^: 536.

##### 2-(2-(4-(4-(4-fluorophenyl)piperazin-1-yl)benzylidene)hydrazineyl)-4-(4-(trifluoromethyl)phenyl)thiazole (**3i**)

Yield: 79%, M.P. = 237.8–240.1 °C, ^1^H-NMR (300 MHz, DMSO-*d_6_*): δ 3.21–3.23 (m, 4H, piperazine), 3.35–3.38 (m, 4H, piperazine), 7.01–7.07 (m, 6H, fluorophenyl, disubstituephenyl), 7.51–7.55 (m, 3H, thiazole, disubstiuephenyl), 7.76 (d, 2H, *J* = 8.4 Hz, disubstituephenyl), 7.96 (s, 1H, −N=CH−), 8.06 (d, 2H, *J* = 8.1 Hz, disubstituephenyl), 12.00 (s, 1H, −NH). ^13^C-NMR (75 MHz, DMSO-*d_6_*): δ = 48.0 (CH_2_), 49.5 (CH_2_), 106.5 (CH), 115.5 (CH), 115.8 (*J_2_* = 21.7 Hz, CH), 118.0 (*J_3_* = 7.6 Hz, CH), 124.8 (q, *J_1_* = 269.9 Hz, C), 125.1 (C), 126.0 (q, *J_3_* = 3.5 Hz, CH), 126.5 (CH), 127.5 (q, *J_2_* = 31.5 Hz, CH), 128.0, (C) 138.9 (C), 142.6 (CH), 148.2 (q, *J_4_* = 1.7 Hz, C), 149.5 (C), 152.0 (C), 156.7 (d, *J_1_* = 234.5 Hz, C), 169.1 (C). ESI-MS [M + H]^+^: 526.

##### 4-(2,4-dimethylphenyl)-2-(2-(4-(4-(4-fluorophenyl)piperazin-1-yl)benzylidene)hydrazineyl)thiazole (**3j**)

Yield: 81%, M.P. = 240.6–243.1 °C. ^1^H-NMR (300 MHz, DMSO-*d_6_*): δ 2.27 (s, 3H, −CH_3_), 2.41 (s, 3H, −CH_3_), 3.19–3.22 (m, 4H, piperazine), 3.34–3.37 (m, 4H, piperazine), 6.81 (s, 1H, thiazole), 7.01–7.06 (m, 7H, fluorophenyl, trisubstituephenyl), 7.50 (d, 2H, *J* = 8.0 Hz, disubstituephenyl), 7.52 (d, 2H, *J* = 8.9 Hz, disubstituephenyl), 7.94 (s, 1H, −N=CH−), 11.83 (s, 1H, −NH). ^13^C-NMR (75 MHz, DMSO-*d_6_*): δ = 21.1 (CH_3_), 21.6 (CH_3_), 48.0 (CH_2_), 49.5 (CH_2_), 105.7 (CH), 115.5 (CH), 115.8 (d, *J_2_* = 21.8 Hz, CH), 118.0 (d, *J_3_* = 7.5 Hz, CH), 125.4 (C), 126.8 (CH), 127.9 (CH), 129.6 (CH), 131.9 (CH), 132.6 (C), 135.5 (C), 137.0 (C), 142.1 (CH), 148.2 (C), 151.1 (C), 151.9 (C), 156.7 (d, *J_1_* = 234.4 Hz, C), 167.8 (C). ESI-MS [M + H]^+^: 486.

##### 4-(2,4-dichlorophenyl)-2-(2-(4-(4-(4-fluorophenyl)piperazin-1-yl)benzylidene)hydrazineyl)thiazole (**3k**)

Yield: 78%, M.P. = 216.2–219.3 °C, ^1^H-NMR (300 MHz, DMSO-*d_6_*): δ 3.22–3.24 (m, 4H, piperazine), 3.36–3.38 (m, 4H, piperazine), 7.02–7.07 (m, 6H, fluorophenyl, disubstituephenyl), 7.36 (s, 1H, thiazole), 7.49 (dd, 1H, *J_1_* = 2.2 Hz, *J_2_* = 8.6 Hz, trisubstituepheyl), 7.53 (d, 2H, *J* = 8.9 Hz, disubstituephenyl), 7.67 (d, 1H, *J* = 2.2 Hz, trisubstituephenyl), 7.91 (d, 1H, *J* = 8.5 Hz, trisubstituephenyl), 7.96 (s, 1H, −N=CH−), 11.96 (s, 1H, −NH). ^13^C-NMR (75 MHz, DMSO-*d_6_*): δ = 47.9 (CH2), 49.6 (CH2), 109.2 (CH), 115.6 (CH), 115.8 (*J_2_* = 21.8 Hz, CH), 118.1 (*J_3_* = 7.5 Hz, CH), 125.3 (C), 127.9 (CH), 128.0 (CH), 130.2 (CH), 132.0 (C), 132.7 (CH), 132.9 (C), 142.5 (CH), 146.3 (C), 147.9 (C), 151.9 (C), 156.9 (*J_1_* = 234.9 Hz, C), 168.0 (C). ESI-MS [M + H]^+^: 526.

### 3.2. Activity Studies

#### 3.2.1. Anticholinesterase Activity Assay

The inhibitory capacity of the obtained compounds on AChE and BChE biological activities was assessed using a modified Ellman’s spectrometric method, as described in our previous studies [34,35,36,37,38]. Donepezil were used as a reference drug. All pipetting procedures in the enzyme inhibition assay were carried out via a robotics system, Biotek Precision XS (Winooski, VT, USA). A BioTek-Synergy H1 microplate reader (Winooski, VT, USA) was used to carry out measurements of percent inhibition at 412 nm, and the IC_50_ values of the selected compounds were performed as previously described.

#### 3.2.2. Monoamine Oxidase Activity Assay

To investigate the MAO-A and MAO-B enzyme inhibition profiles of the synthesized compounds, the fluorometric enzyme inhibition assay was carried out, as previously defined by us [30,38,39,40]. Ampliflu™ Red (10-Acetyl-3,7-dihydroxyphenoxazine) was used as a fluorescence reagent in this assay. All reagents and enzymes (Ampliflu™ Red, peroxidase from horseradish, hMAO-A, hMAO-B, H2O2, tyramine hydrochloride, moclobemide and selegiline) were supplied by Sigma-Aldrich (Steinheim, Germany).

#### 3.2.3. Kinetic Studies of Enzyme Inhibition

In order to determine the type of inhibition on AChE, enzyme kinetic studies were carried out for compound **3c**. The enzyme kinetic assay was performed by means of Ellman’s method, in keeping with the previous studies reported by our research group [34,35,36,37,38].

### 3.3. Molecular Docking Studies

Docking studies using the in silico procedure were carried out for compound **3c** in order to determine the binding modes on the AChE enzyme active site. The X-ray crystal structure (PDB ID: 4EY7) [28] of AChE from the Protein Data Bank server (www.pdb.org) was provided, and the docking procedure was performed as described in our previous work [34,35,36,37,38].

### 3.4. Prediction of BBB Permeability

The BBB permeability of the compounds was evaluated by an online BBB Predictor [33].

## 4. Conclusions

AD is a progressive neurodegenerative disease related to the presence of extracellular neuritic plaques, or senile confusion in the intracellular neurofibrillary areas of the brain. Currently, there are 35 million people with AD dementia in the world, and this number will increase to approximately 100 million globally by 2050 if treatments are not found. In the present work, a series of novel thiazole-piperazine analogs (**3a**–**k**) were designed, synthesized and evaluated as inhibitors against AChE and BChE. Compounds **3a**, **3c** and **3i** (IC_50_ 0.0496, 0.0317 and 0.0287 µM against AChE, respectively) are the most potent ones in the series. None of them show strong affinity for BChE. So as to elucidate their in silico properties and the type of inhibition, binding studies and enzyme kinetics were performed for these compounds. The kinetic studies suggested that these compounds were reversible and mixed type enzyme inhibitors. 

Donepezil can bind strongly to the enzyme active region owing to its dual binding sites. The 5,6-dimethoxyindanone moiety binds to the peripheral anionic site (PAS) of the enzyme active region by interacting with the Trp286 and Phe295 amino acid residues. The rest of the structure, the benzylpiperidine group, binds to the catalytic anionic site (CAS) by interacting with Trp86 [34]. In keeping with the molecular docking studies, compound 3c displayed effective AChE inhibition capability based on multiple binding patterns in the PAS and CAS of AChE. As already known, donepezil was able to bind strongly to the enzyme active region owing to its dual binding sites.

## Figures and Tables

**Table 1:** Inhibition (%) of compounds **3a**–**k** against AChE and butyrylcholinesterase (BChE) enzymes.

Comp.	AChE % Inhibition		BChE % Inhibition		AChE IC_50_ (µM)	Comp.	AChE % Inhibition		BChE % Inhibition		AChE IC_50_ (µM)
	10^−3^ M	10^−4^ M	10^−3^ M	10^−4^ M			10^−3^ M	10^−4^ M	10^−3^ M	10^−4^ M	
**3a**	84.20 ± 0.89	70.58 ± 0.75	21.53 ± 0.38	19.32 ± 0.28	0.0496 ± 0.002	**3g**	64.55 ± 0.67	37.80 ± 0.60	38.54 ± 0.35	20.25 ± 0.35	-
**3b**	75.28 ± 1.05	45.90 ± 0.88	37.40 ± 0.67	25.34 ± 0.58	-	**3h**	75.33 ± 1.06	47.12 ± 0.74	40.65 ± 0.68	35.52 ± 0.41	-
**3c**	88.45 ± 1.10	72.20 ± 0.97	27.96 ± 0.43	21.64 ± 0.65	0.0317 ± 0.001	**3i**	80.70 ± 1.02	62.79 ± 0.82	45.35 ± 0.52	35.36 ± 0.39	0.2158 ± 0.010
**3d**	76.70 ± 1.12	38.55 ± 0.76	35.38 ± 0.49	31.85 ± 0.56	-	**3j**	53.75 ± 0.62	26.90 ± 0.55	31.88 ± 0.47	25.66 ± 0.49	-
**3e**	71.20 ± 0.90	42.32 ± 0.63	31.79 ± 0.61	24.86 ± 0.38	-	**3k**	35.66 ± 0.57	20.40 ± 0.30	28.59 ± 0.25	20.96 ± 0.61	-
**3f**	78.40 ± 1.04	48.15 ± 0.87	28.19 ± 0.42	20.45 ± 0.35	-	**Donepezil**	99.37 ± 1.16	98.64 ± 1.09	-	-	0.0287 ± 0.005

**Table 2:** Inhibition (%) of compounds **3a**–**3k** against MAO-A and MAO-B enzymes.

Comp.	MAO-A % Inhibition		MAO-B % Inhibition		MAO-B IC_50_ (µM)	Comp.	MAO-A % Inhibition		MAO-B% Inhibition		MAO-B IC_50_ (µM)
	10^−3^ M	10^−4^ M	10^−3^ M	10^−4^ M			10^−3^ M	10^−4^ M	10^−3^ M	10^−4^ M	
**3a**	38.20 ± 0.95	20.15 ± 0.63	78.25 ± 1.05	70.23 ± 0.99	2.107 ± 0.086	**3g**	48.23 ± 0.97	30.21 ± 0.50	68.12 ± 1.12	41.75 ± 0.86	-
**3b**	44.21 ± 1.08	25.75 ± 0.80	60.82 ± 1.11	46.7 ± 0.98	-	**3h**	34.17 ± 0.58	22.19 ± 0.47	68.92 ± 1.25	39.11 ± 0.63	-
**3c**	38.65 ± 0.58	21.79 ± 0.55	85.12 ± 1.00	70.26 ± 0.85	1.015 ± 0.042	**3i**	38.19 ± 0.78	22.75 ± 0.54	59.08 ± 0.77	40.25 ± 0.58	-
**3d**	50.98 ± 0.97	39.12 ± 0.71	75.66 ± 1.06	65.17 ± 0.97	5.204 ± 0.153	**3j**	48.21 ± 0.88	40.23 ± 0.52	69.20 ± 1.20	40.75 ± 0.92	-
**3e**	47.22 ± 0.88	23.50 ± 0.60	58.11 ± 0.90	43.08 ± 0.71	-	**3k**	35.20 ± 0.48	19.14 ± 0.40	55.09 ± 0.92	34.17 ± 0.81	-
**3f**	30.11 ± 0.47	19.28 ± 0.41	57.33 ± 1.03	39.20 ± 0.79	-	**Moclobemide**	91.25 ± 2.8	78.20 ± 2.55	-	-	-
						**Selegiline**	-	-	98.21 ± 2.05	94.12 ± 1.96	0.040 ± 0.002

## Supplementary Materials

The following are available online, Figure S1: 2-amino-4-substituted thiazole as a pharmacophore for acetylcholinesterase (AChE), Figure S2: Some AchE inhibitors, including a benzylamine moiety, Figure S3: General structure of target compounds, Figure S4: (**A**) Lineweaver–Burk plots for the inhibition of AChE by compound **3c**. [S], substrate concentration (μM); V, reaction velocity (abs/min)^−1^. (**B**) Secondary plot for the calculation of steady-state inhibition constant (K_i_) of compound **3c**. Ki was calculated as 0.0197 μM, Figure S5: The interacting mode of compound **3c** in the active region of AChE. The inhibitor, colored with yellow, and the important residues, colored with purple, in the active site of the enzyme are presented by a tube model, Table S1: Inhibition (%) of compounds **3a**–**k** against AChE and butyrylcholinesterase (BChE) enzymes, Table S2: Inhibition (%) of compounds **3a**–**3k** against MAO-A and MAO-B enzymes.
