# Supplementary material for: Synthesis and AChE Inhibitory Activity of Novel Thiazolylhydrazone Derivatives
Source: Molecules. 2019 Jun 28;24(13):2392. doi: 10.3390/molecules24132392 (PMC6651548; doi:10.3390/molecules24132392)
Supplement: Supplementary file 1 [file molecules-24-02392-s001.pdf]

SUPPORTING INFORMATION

Synthesis And AChE Inhibitory Activity of Novel Thiazole-Piperazine

Derya Osmaniye[,1,2], Begüm Nurpelin Sağlık[1,2], Ulviye Acar Çevik[1,2], , Serkan Levent[1,2], Betül Kaya Çavuşoğlu[1], Yusuf Özkay[1,2,*], Zafer Asım Kaplancıklı[1], Gülhan Turan[1]

[a]*Department of Pharmaceutical Chemistry, Faculty of Pharmacy, Anadolu University, 26470 Eskişehir, Turkey*

[b]*Doping and Narcotic Compounds Analysis Laboratory, Faculty of Pharmacy, Anadolu University, 26470 Eskişehir, Turkey*

* Corresponding author.

E-mail address: yozkay@anadolu.edu.tr (Y. Özkay).

Tel: +90-222-3350580/3779 *Fax*: +90-222-3350750.

Address: Anadolu University, Faculty of Pharmacy, Department of Pharmaceutical Chemistry, 26470, Eskişehir, Turkey.

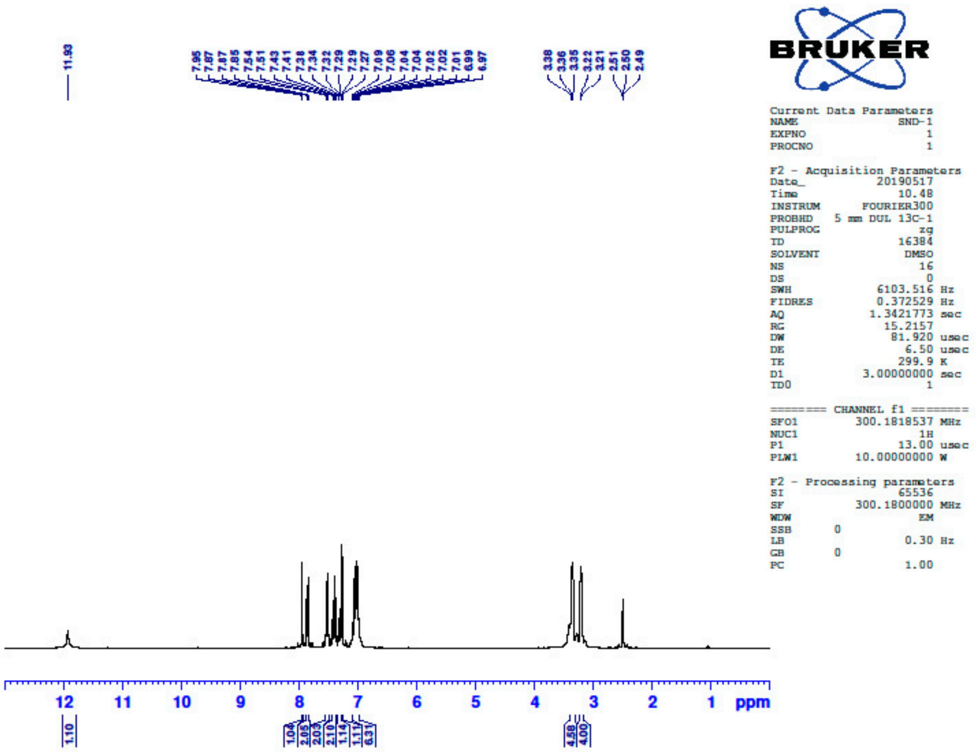

Spectra 1. ^{1}H-NMR spectra of compound **3a**

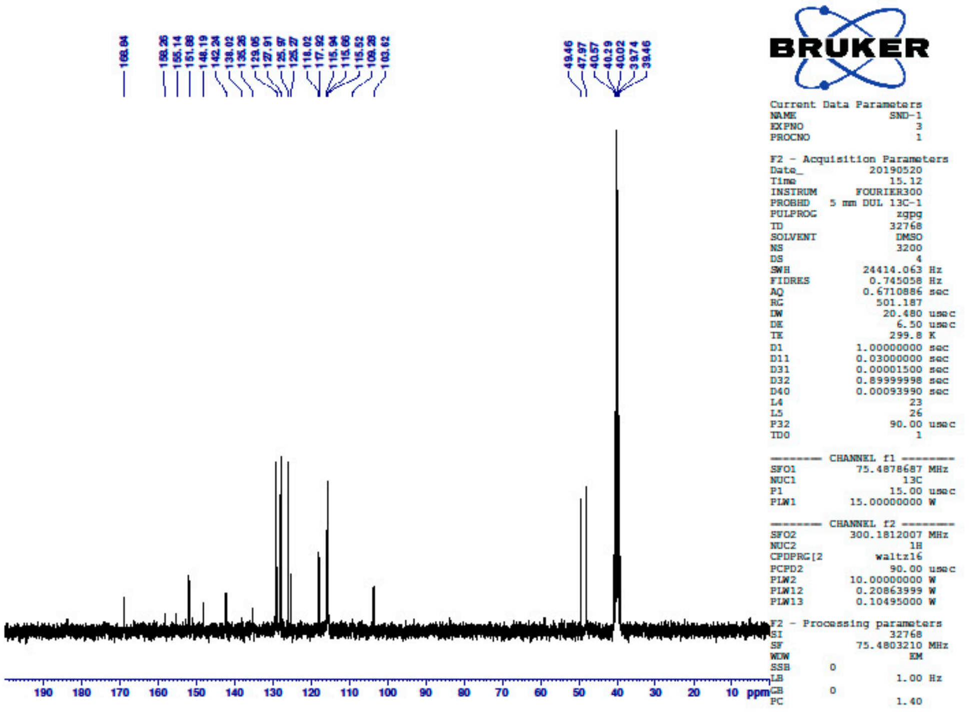

Spectra 2. ^{13}C-NMR spectra of compound **3a**

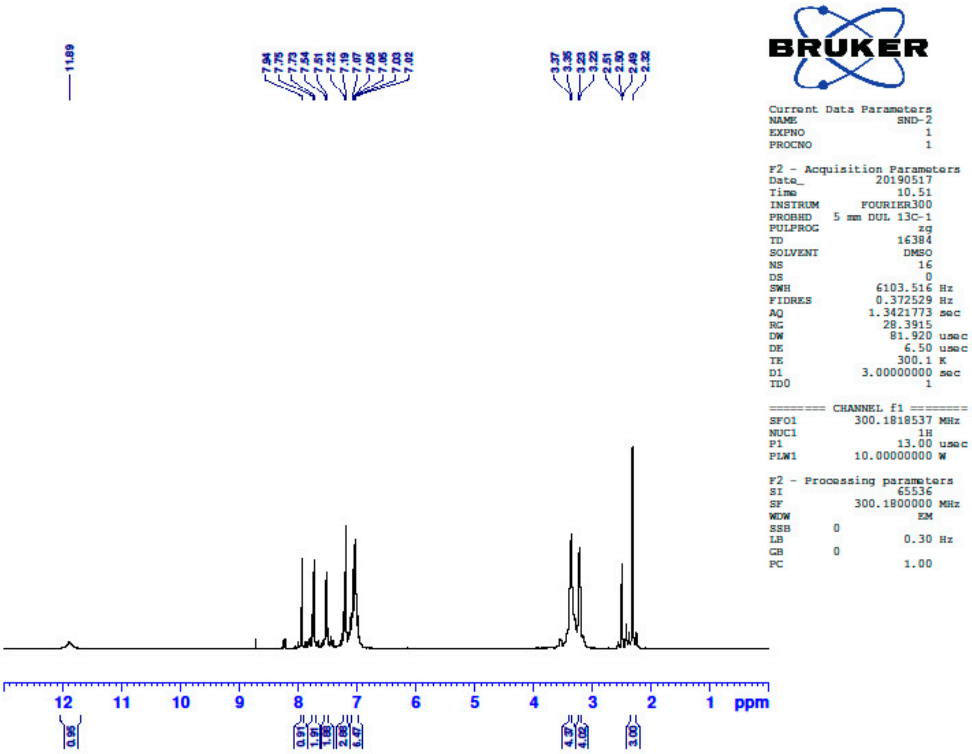

Spectra 3. ^{1}H-NMR spectra of compound **3b**

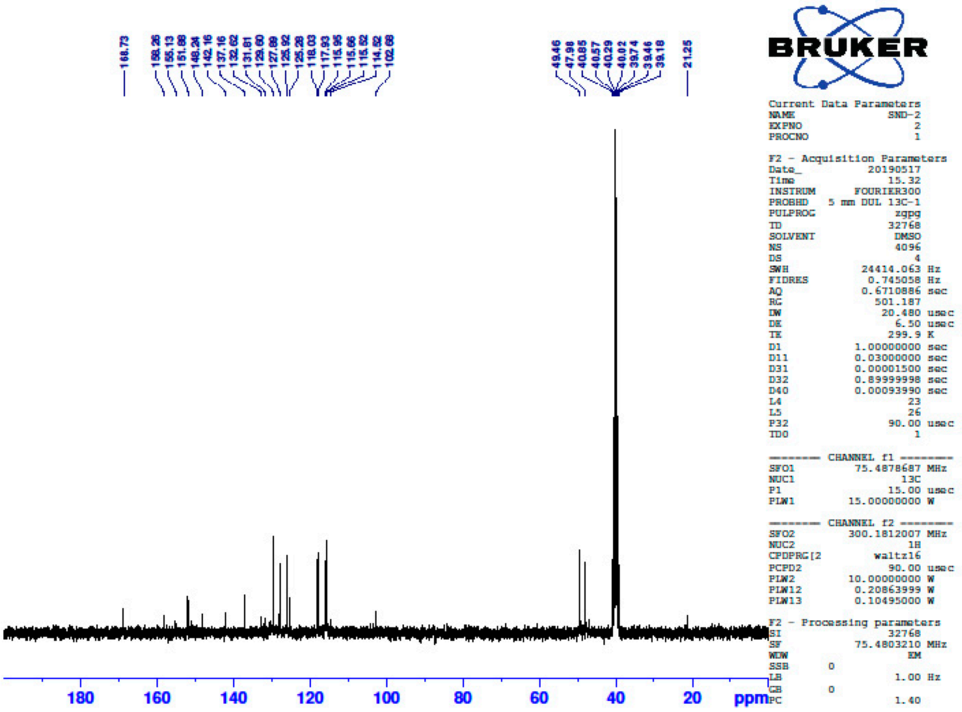

Spectra 4. ^{13}C-NMR spectra of compound **3b**

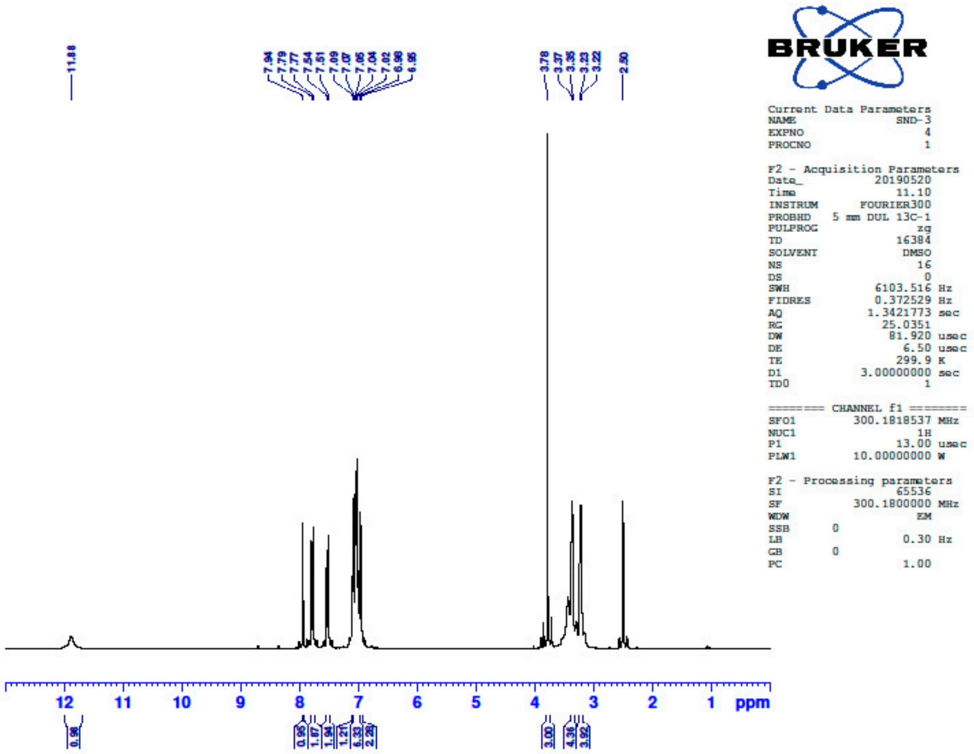

Spectra 5. ^{1}H-NMR spectra of compound **3c**

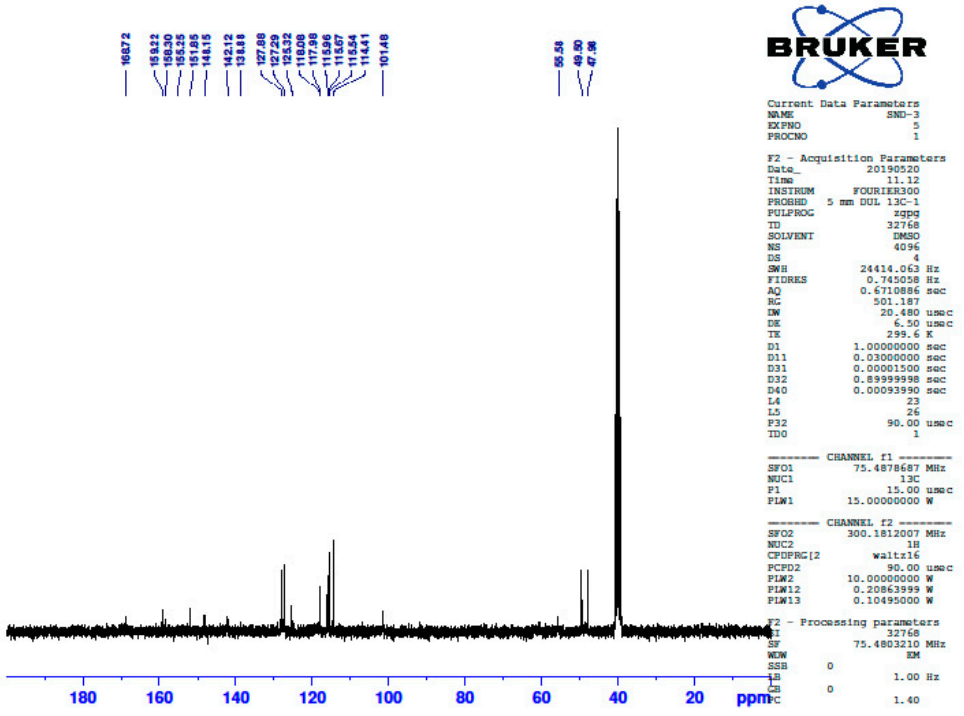

Spectra 6. ^{13}C-NMR spectra of compound **3c**

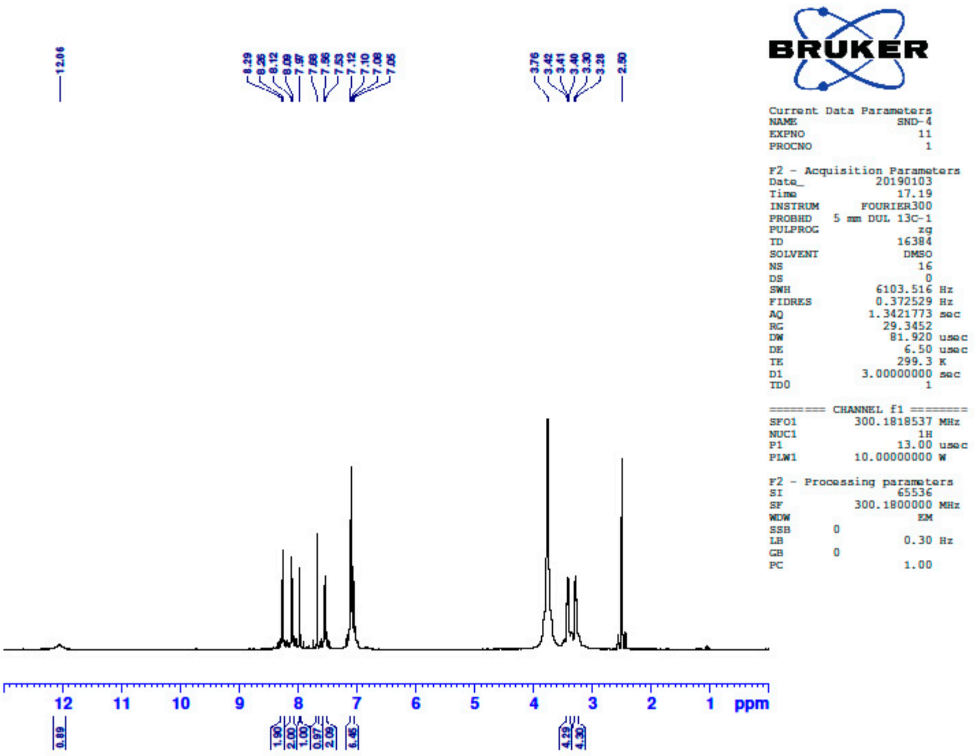

Spectra 7. ^{1}H-NMR spectra of compound **3d**

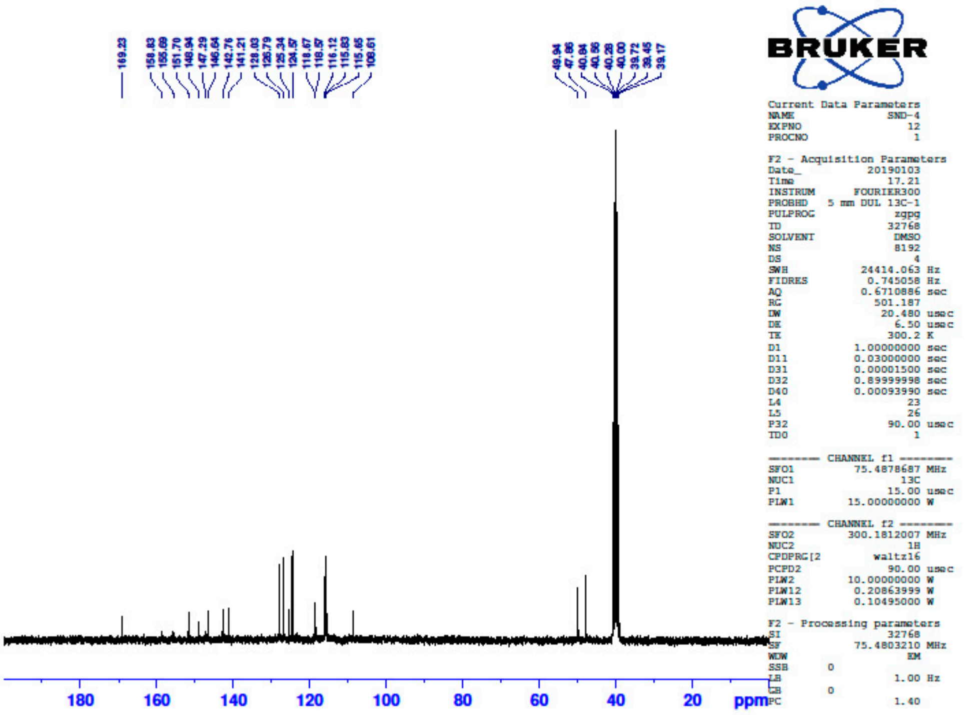

Spectra 8. ^{13}C-NMR spectra of compound **3d**

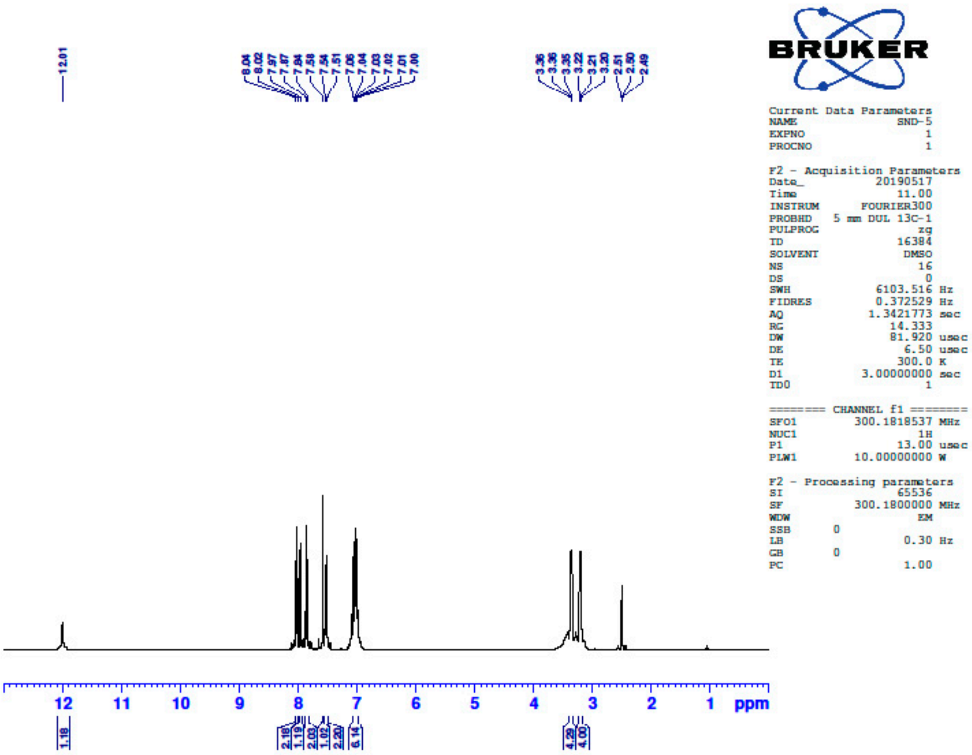

Spectra 9. ^{1}H-NMR spectra of compound **3e**

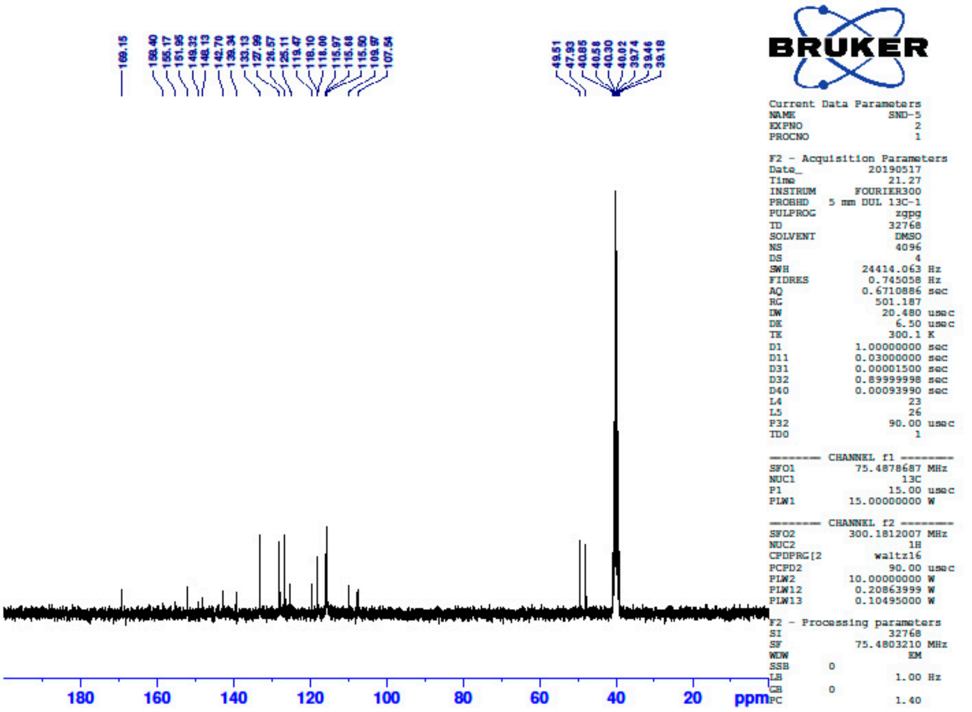

Spectra 10. ^{13}C-NMR spectra of compound **3e**

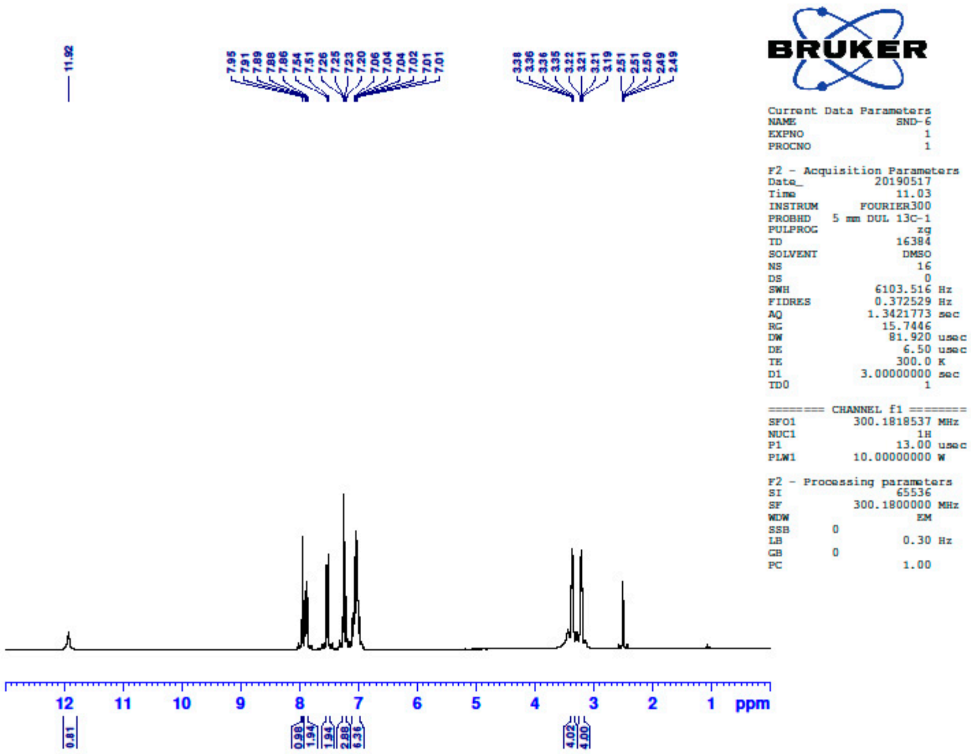

Spectra 11. ^{1}H-NMR spectra of compound **3f**

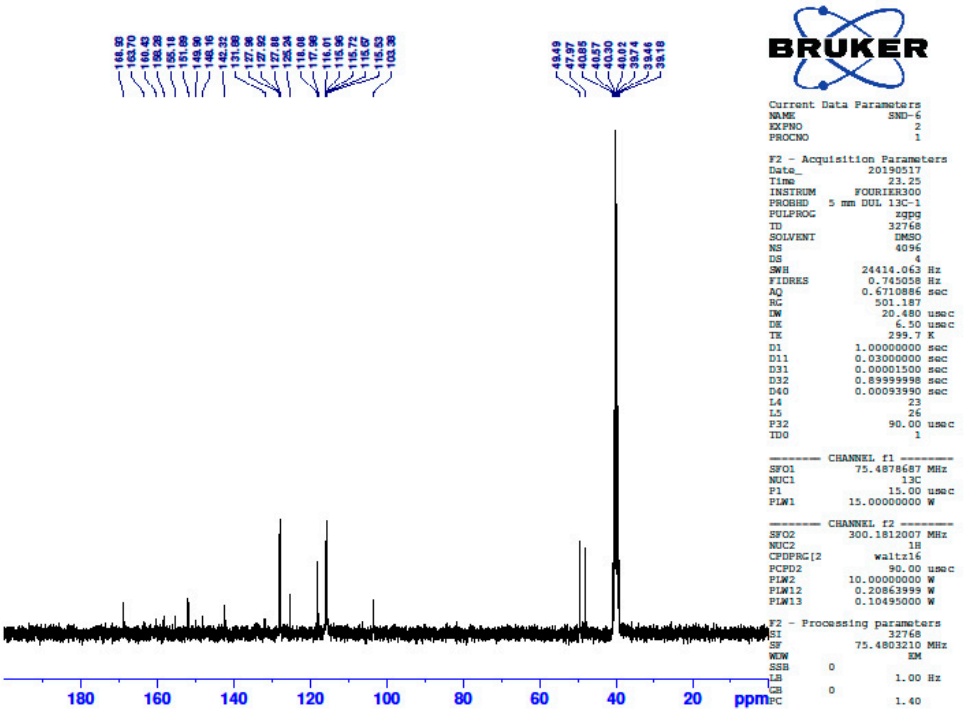

Spectra 12. ^{13}C-NMR spectra of compound **3f**

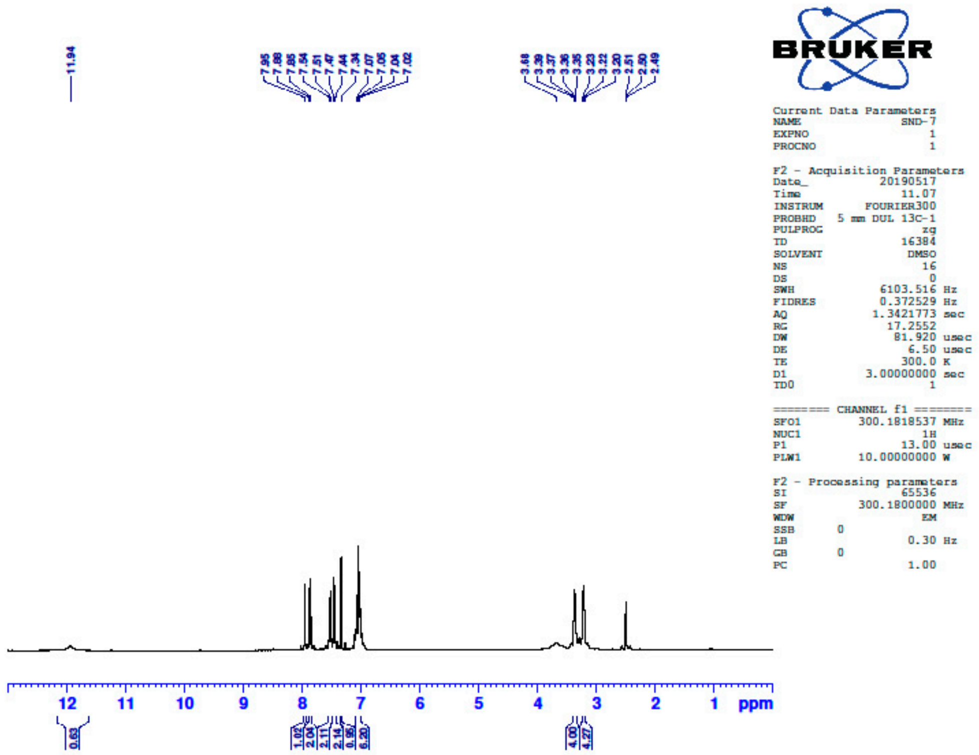

Spectra 13. ^{1}H-NMR spectra of compound **3g**

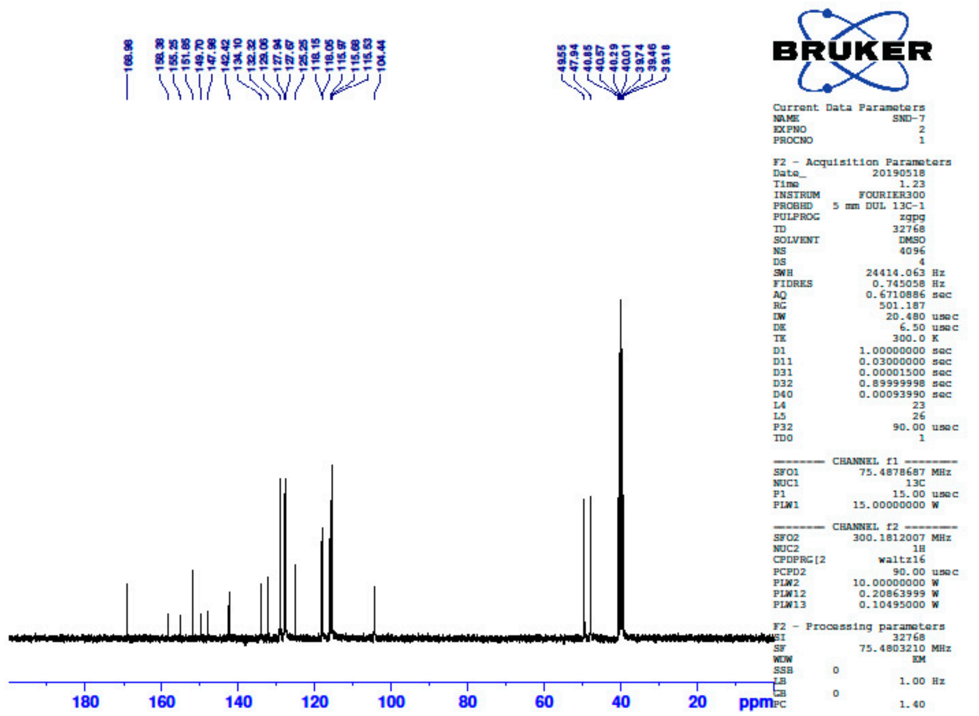

Spectra 14. ^{13}C-NMR spectra of compound **3g**

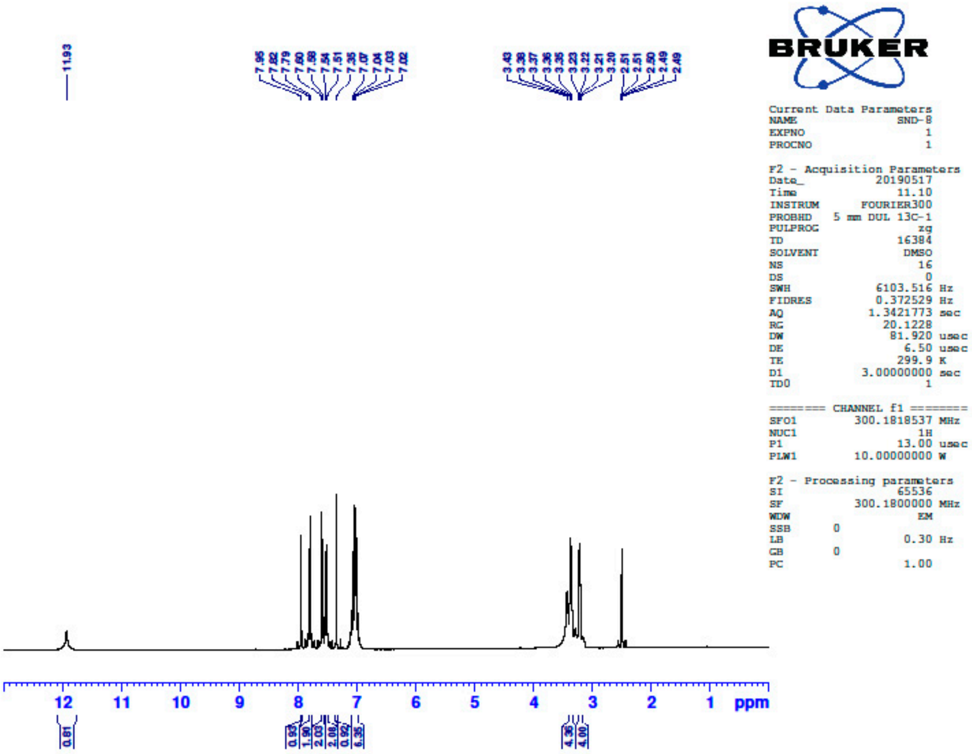

Spectra 15. ^{1}H-NMR spectra of compound **3h**

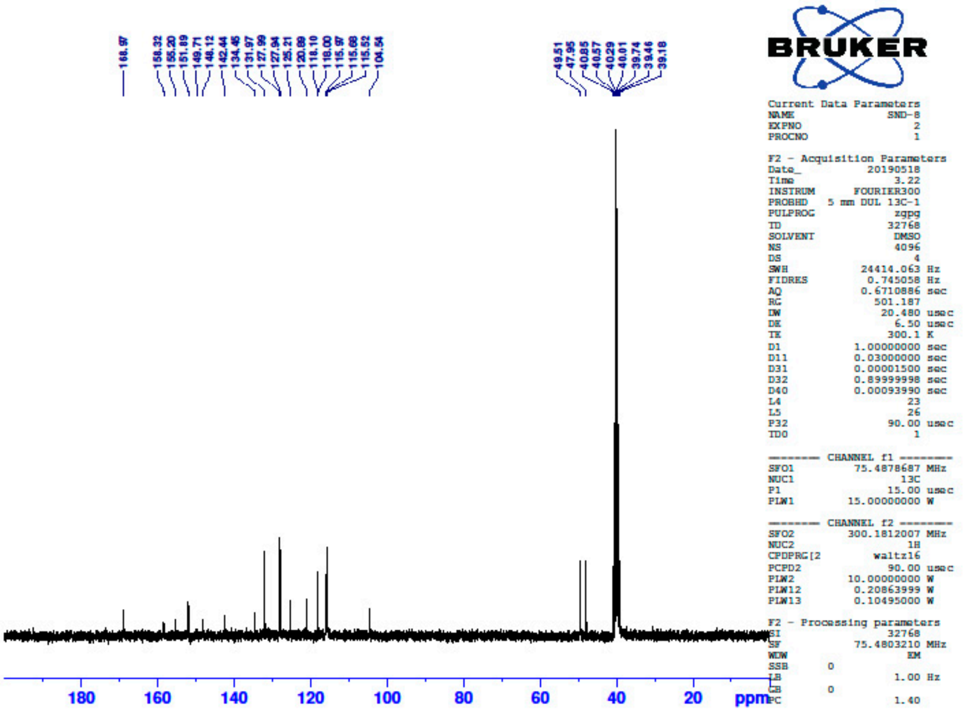

Spectra 16. ^{13}C-NMR spectra of compound **3h**

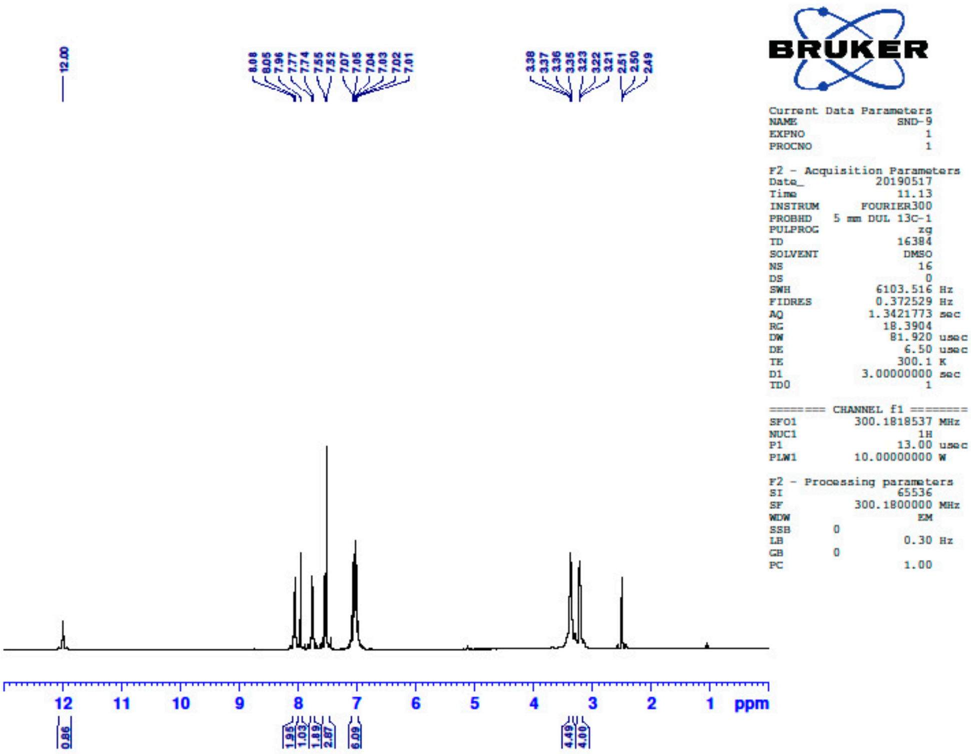

Spectra 17. ^{1}H-NMR spectra of compound **3i**

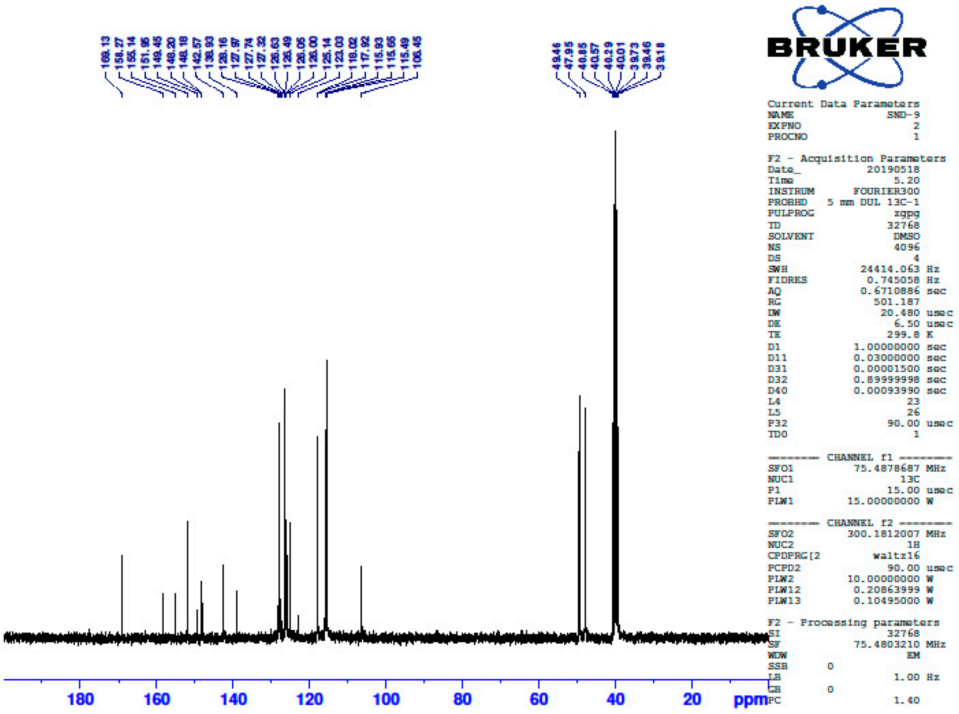

Spectra 18. ^{13}C-NMR spectra of compound **3i**

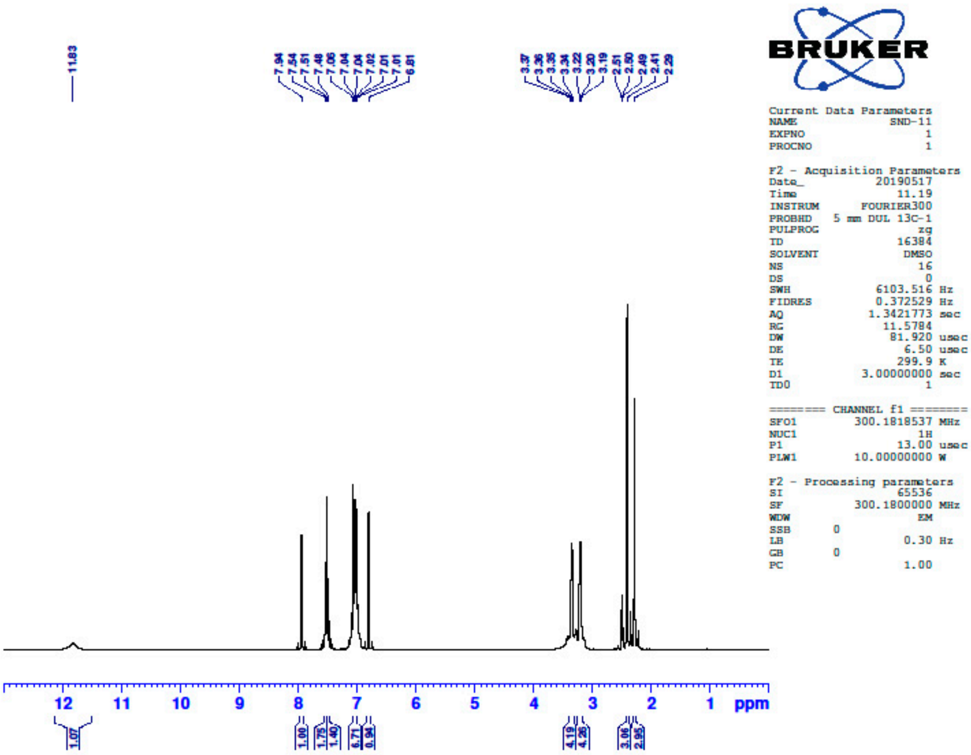

Spectra 19. ^{1}H-NMR spectra of compound **3j**

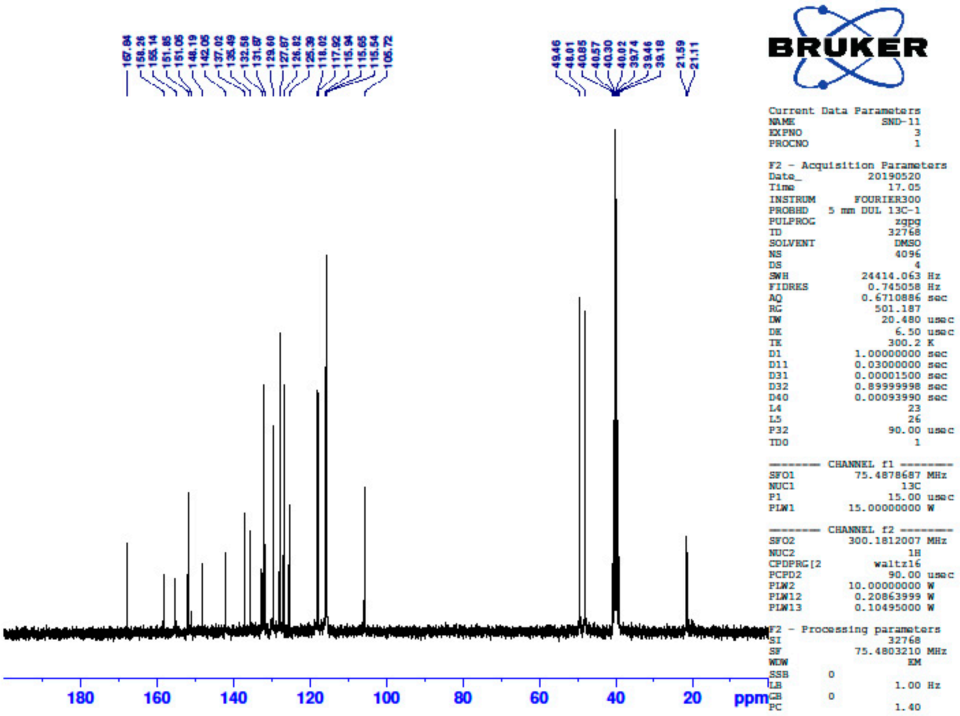

Spectra 20. ^{13}C-NMR spectra of compound **3j**

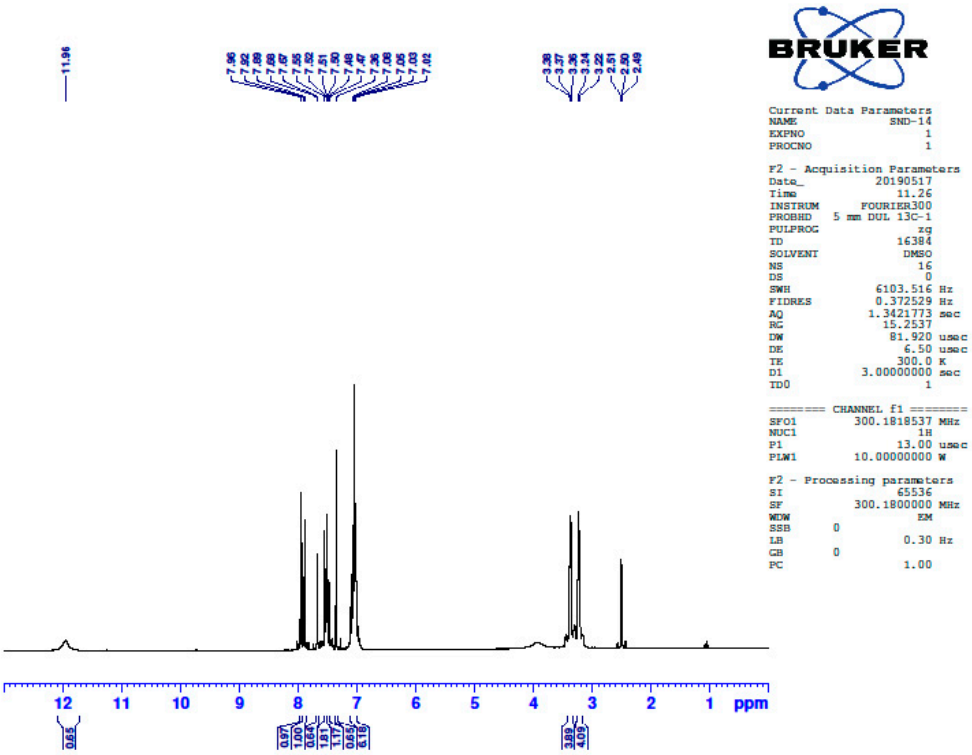

Spectra 21. ^{1}H-NMR spectra of compound **3k**

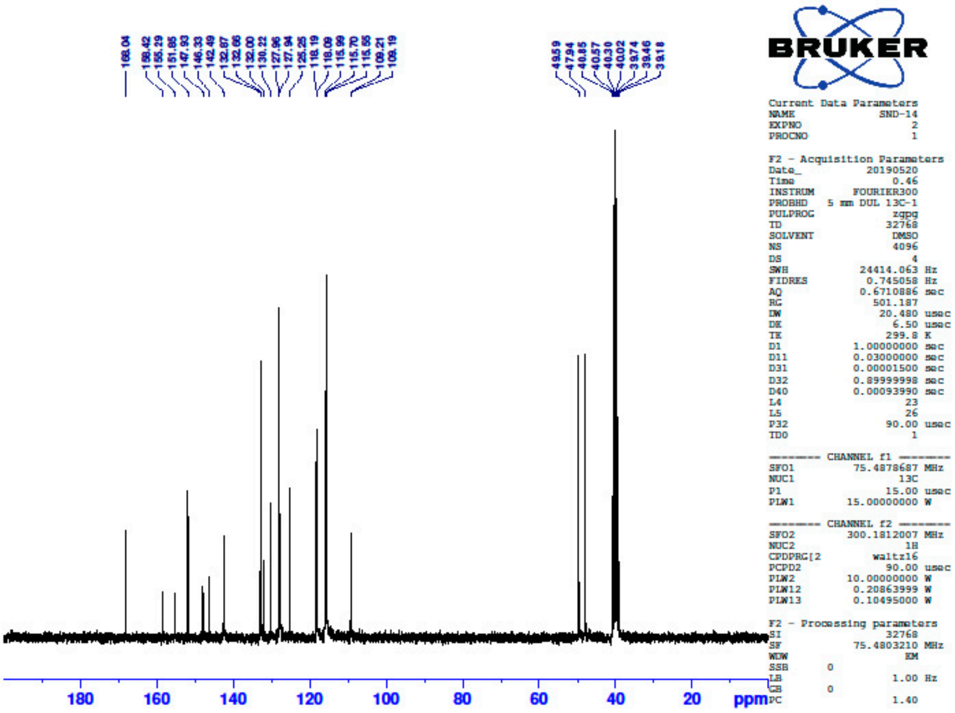

Spectra 22. ^{13}C-NMR spectra of compound **3k**
